# Supplementary material for: Multi‐dataset identification of innovative feature genes and molecular mechanisms in keratoconus
Source: J Cell Mol Med. 2024 Sep 19;28(18):e70079. doi: 10.1111/jcmm.70079 (PMC11412914; doi:10.1111/jcmm.70079)
Supplement: Supplementary file 2 — Material S2. [file JCMM-28-e70079-s002.zip › 7.GSEA analysis/2.ATOH7/KEGG_BUTANOATE_METABOLISM.html]

Details for gene set KEGG\_BUTANOATE\_METABOLISM[GSEA]

|  || Dataset | ATOH7.group.cls#h\_versus\_l |
| Phenotype | group.cls#h\_versus\_l |
| Upregulated in class | h |
| GeneSet | KEGG\_BUTANOATE\_METABOLISM |
| Enrichment Score (ES) | 0.5293808 |
| Normalized Enrichment Score (NES) | 1.5598857 |
| Nominal p-value | 0.029850746 |
| FDR q-value | 0.26686797 |
| FWER p-Value | 0.681 |
Table: GSEA Results Summary

  

Fig 1: Enrichment plot: KEGG\_BUTANOATE\_METABOLISM      
 Profile of the Running ES Score & Positions of GeneSet Members on the Rank Ordered List

  

| SYMBOL | TITLE | RANK IN GENE LIST | RANK METRIC SCORE | RUNNING ES | CORE ENRICHMENT || 1 | ALDH2 | na | 221 | 0.659 | 0.1386 | Yes |
| 2 | ACSM3 | na | 321 | 0.616 | 0.2713 | Yes |
| 3 | AKR1B10 | na | 422 | 0.570 | 0.3938 | Yes |
| 4 | ALDH5A1 | na | 1652 | 0.373 | 0.4403 | Yes |
| 5 | BDH2 | na | 2886 | 0.287 | 0.4678 | Yes |
| 6 | OXCT2 | na | 3879 | 0.241 | 0.4921 | Yes |
| 7 | GAD1 | na | 4303 | 0.225 | 0.5294 | Yes |
| 8 | ALDH9A1 | na | 7741 | 0.125 | 0.4577 | No |
| 9 | ALDH1B1 | na | 7850 | 0.122 | 0.4814 | No |
| 10 | EHHADH | na | 8475 | 0.109 | 0.4873 | No |
| 11 | OXCT1 | na | 12428 | 0.047 | 0.3837 | No |
| 12 | HADH | na | 13799 | 0.033 | 0.3514 | No |
| 13 | ALDH7A1 | na | 14436 | 0.026 | 0.3389 | No |
| 14 | ACSM5 | na | 15639 | 0.016 | 0.3076 | No |
| 15 | ACAT1 | na | 15830 | 0.014 | 0.3053 | No |
| 16 | ACADS | na | 16641 | 0.008 | 0.2836 | No |
| 17 | HADHA | na | 16925 | 0.005 | 0.2766 | No |
| 18 | ECHS1 | na | 16985 | 0.005 | 0.2759 | No |
| 19 | PDHA2 | na | 17634 | -0.001 | 0.2574 | No |
| 20 | PDHB | na | 17788 | -0.002 | 0.2535 | No |
| 21 | BDH1 | na | 18784 | -0.011 | 0.2272 | No |
| 22 | ALDH3A2 | na | 20819 | -0.030 | 0.1752 | No |
| 23 | PDHA1 | na | 21078 | -0.032 | 0.1749 | No |
| 24 | ACSM2A | na | 22061 | -0.044 | 0.1561 | No |
| 25 | HMGCS2 | na | 23196 | -0.057 | 0.1361 | No |
| 26 | L2HGDH | na | 23700 | -0.065 | 0.1359 | No |
| 27 | ABAT | na | 23756 | -0.066 | 0.1489 | No |
| 28 | ACAT2 | na | 23817 | -0.067 | 0.1618 | No |
| 29 | HMGCS1 | na | 24316 | -0.074 | 0.1638 | No |
| 30 | AACS | na | 24720 | -0.081 | 0.1700 | No |
| 31 | HMGCL | na | 25099 | -0.087 | 0.1782 | No |
| 32 | ACSM1 | na | 25857 | -0.101 | 0.1786 | No |
| 33 | ACSM4 | na | 25955 | -0.103 | 0.1984 | No |
| 34 | GAD2 | na | 31008 | -0.247 | 0.1072 | No |
Table: GSEA details [plain text format]

  

Fig 2: KEGG\_BUTANOATE\_METABOLISM      
 Blue-Pink O' Gram in the Space of the Analyzed GeneSet

  

Fig 3: KEGG\_BUTANOATE\_METABOLISM: Random ES distribution      
 Gene set null distribution of ES for **KEGG\_BUTANOATE\_METABOLISM**
